# Beetling the heat – the diurnal Namib Desert beetle *Onymacris plana* cools by running

**DOI:** 10.1242/jeb.250379

**Published:** 2025-08-14

**Authors:** Carole S. Roberts, Elizabeth L. McClain, Mary K. Seely, Duncan Mitchell, Victoria L. Goodall, Joh R. Henschel

**Affiliations:** ^1^ Gobabeb Namib Research Institute, Walvis Bay 13013, Namibia; ^2^ Brain Function Research Group, School of Physiology, University of the Witwatersrand, Braamfontein 2000, Johannesburg, South Africa; ^3^ School of Human Sciences, University of Western Australia, Perth WA 6009, Australia; ^4^ Department of Statistics, Nelson Mandela University, Gqeberha 6031, South Africa; ^5^ South African Environmental Observation Network, Kimberley 8301, South Africa; ^6^ Centre for Environmental Management, University of the Free State, Bloemfontein 9301, South Africa

**Keywords:** Tenebrionidae, Locomotion, Thermoregulation, Radiant flux, Convection, Dune

## Abstract

*Onymacris plana* (Coleoptera: Tenebrionidae) is a black beetle that runs at high speed for a pedestrian insect in direct solar radiation in the Namib Desert, a behaviour expected to impose potentially lethal body temperature within minutes. We measured the body temperature of beetles active in their natural habitat using fine thermocouples inserted into the prothorax. The measurements revealed that when beetles sprinted in conditions of low wind, high radiation and moderate ambient temperature, their body temperature dropped rather than rose. The effect depended on convective cooling and efficient locomotion, i.e. sprinting with low energy expenditure. We confirmed the convection effect in the laboratory by exposing beetles to combinations of radiation, air temperature and wind speed comparable to those found in the Namib Desert and simulating the forced convection of running in a headwind. Under these simulated conditions, peak radiation caused the temperature of stationary male beetles to rise at about 6°C min^−1^ and females at almost 4°C min^−1^. However, in wind-calm conditions at peak radiation, the convection of simulated running dropped the equilibrium body temperature of live beetles by about 13°C. We believe that ours is the first report of exercise-induced cooling in a pedestrian animal and that *O. plana*'s diurnal lifestyle depends on that exercise-induced cooling.

## Teaser

**Highlighted Article:** Efficient running enables a diurnal Namib Desert dune beetle to cool convectively by headwind during hot, wind-still conditions.

## INTRODUCTION

The flightless beetle *Onymacris plana* (Péringuey) (Coleoptera: Tenebrionidae) is endemic to the Namib Dune Sea in southwestern Namibia (Penrith, 1975). It inhabits sparsely vegetated dunes, which are subjected to intense solar radiation that generates surface temperatures exceeding 50°C during 8 months of the year (Lancaster et al., 1984). The beetles forage by day, avoiding nocturnal predators (Polis et al., 1998; Seely, 1985). Individuals can travel several kilometres daily (Roer, 1975). They feed on wind-blown detritus, predominantly in open, unshaded places or at isolated shady patches, sprinting from one patch to the next over distances that can exceed 100 m, sometimes stopping to feed. They spend many hours on the sand surface (Holm and Edney, 1973), with males spending longer periods while searching for mating opportunities (Enders et al., 1998; Polis et al., 1998; Rasmussen et al., 1991), even when the sand surface temperature exceeds 50°C. Their metabolic rate is almost independent of body temperature between 20 and 40°C (Lease et al., 2014; Roberts, 1991), and their preferred body temperature of 39°C is higher than that of other sympatric beetles (Roberts et al., 1991). Any animals that are active diurnally on the Namib dune surface must restrict heat gain or augment heat loss to maintain their body temperature below lethal limits (Mitchell et al., 2024); thermoregulatory behaviour is crucial to their survival (Mitchell et al., 2020). They can escape intolerable surface conditions by seeking shade under sparse vegetation or by swimming into the sand underfoot (Heinrich, 1993; Holm and Edney, 1973; Seely et al., 1988), except during extreme heat waves. During heatwaves, when the temperature of the top 5 cm of sand can exceed 64°C (e.g. in March 1987, J.R.H. personal observations), surface-active beetles cannot escape by digging, and many die.

*Onymacris plana* is distinct among Namib tenebrionids for the speed at which it runs and its peculiar discoid shape. Its large horizontal surface area exposes the beetle to high radiant heat loads, which are intensified by its black colouration (Henwood, 1975a; McClain et al., 1991). Nevertheless, spot measurements of prothoracic temperatures of individuals caught running in the open were no higher than those of individuals standing in the shade, indicating that heat loading from radiation and exercise can be offset (Nicolson et al., 1984). At the hottest part of the day, dorsal and ventral wax secretions enhance the reflectance of solar radiation (McClain et al., 1991), though the elytral surface temperature can reach 60°C (Henwood, 1975b). The beetle's lethal body core temperature is 51°C (Edney, 1971a), which falls within the range of other desert insects (Bennett et al., 2021; Edney, 1971a; Gehring and Wehner, 1995; Marsh, 1985b; Roberts et al., 1991; Seely et al., 1988). *Onymacris plana* is unique in its running speed of 0.9–1.2 m s^−1^, making it the swiftest Namib beetle (Nicolson et al., 1984). That maximum speed is the same as that of the American cockroach *Periplaneta americana*, with similar body mass, which is regarded as ‘one of the fastest invertebrates ever studied’ (Full and Tu, 1991, p. 215), though the Australian tiger beetle *Rivacindela hudsoni*, also similar in size to *O. plana*, can reach 2.5 m s^−1^ (Kamoun and Hogenhout, 1996).

**Fig. 1.**
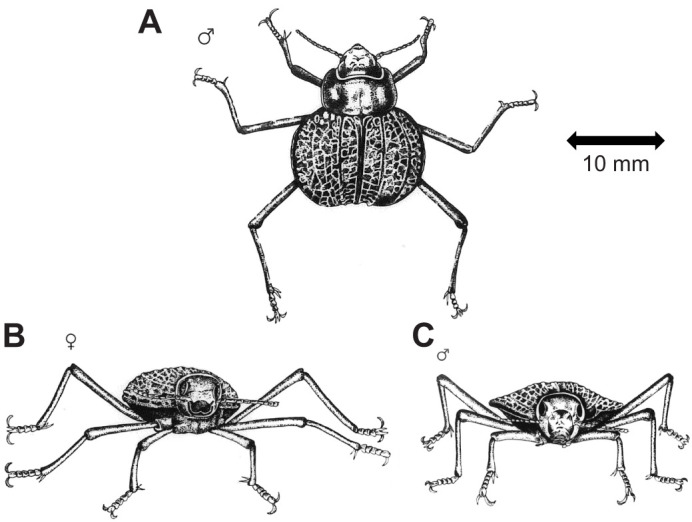
**Body shapes of *Onymacris plana*.** (A) Male dorsal view. (B) Female frontal view. (C) Male frontal view. Artwork by Valerie Myburgh, University of the Witwatersrand.

**Fig. 2.**
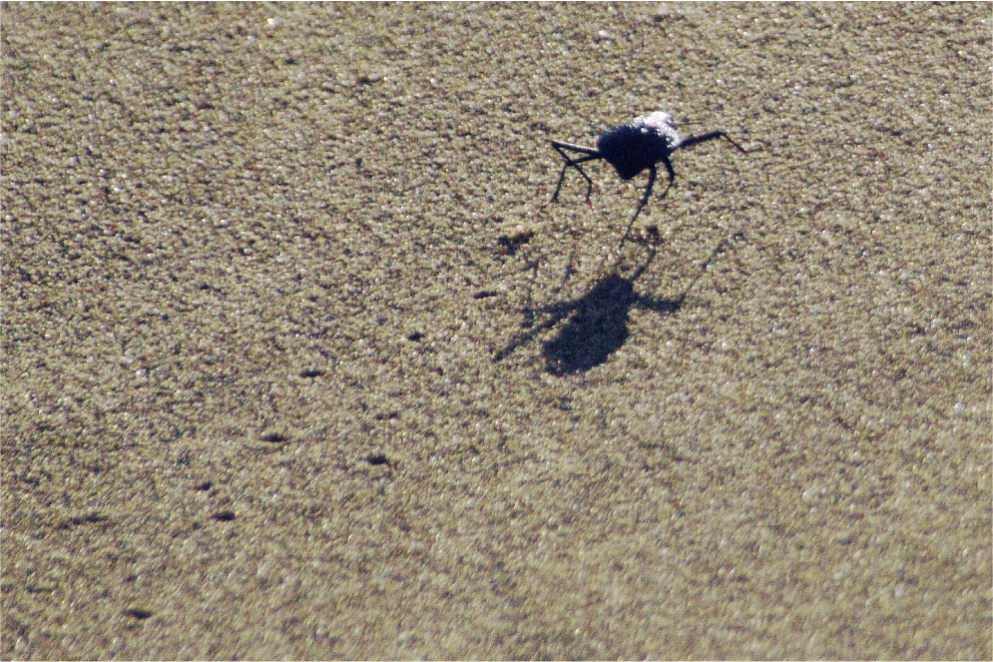
**A sprinting *O. plana* male elevating its body above the sand surface.** Photo credit: J.R.H.

The discoid shape of *O. plana* arises because the beetle is dorsoventrally flattened, with fused elytra that flare out laterally, giving the insect a streamlined appearance (Fig. 1). This shape is developed well only in male *O. plana*. Though it may have other advantages (Enders et al., 1998), the discoid shape is purported to act as an aerofoil that generates aerodynamic lift (Bartholomew et al., 1985) (Fig. 2). In cockroaches, which do not have the discoid shape, the effect of lift on the energetics of running is small (Full and Koehl, 1993). We postulate that the discoid shape would also enhance convective heat transfer due to the increased ratio of surface area to volume and the narrower vertical diameter (Mitchell, 1974), which would be especially important in males, which are exposed more than are females. Among tenebrionid beetles, male *O. plana* also have disproportionately long legs for their mass. This leg morphology would give them additional competitive advantages in running and elevation above hot surfaces, as is the case for Saharan desert ants, *Cataglyphis* spp. (Tross et al., 2021).

Convective cooling can be a major route of heat loss for small insects and can offset heat gain, as observed in many flying insects (Heinrich, 1981). We addressed the possibility that *O. plana* regulate their body temperature by running. The convection induced by body movement may alleviate hyperthermia associated with exercise in the heat, including in humans (Galimidi et al., 1979), and enhance convective heat loss in cold environments (Best, 1982). However, we take it a step further. We propose that running actually may reverse exercise hyperthermia and decrease the beetle's body temperature. We posit that convective cooling offsets metabolic and radiant heat gains such that, counter-intuitively, the beetles might be less at risk of hyperthermia while running than while stationary in the sun. Our hypothesis depends on the near-surface air temperature in the Namib Desert being lower than the sand surface temperature when solar radiation is high (Marsh, 1985b; Seely et al., 1990). We explored whether substantial cooling could be induced by increased air movement across the body surface by running in high radiation. Initially, we conducted field studies in the natural environment, where we measured the body temperature of beetles while sprinting compared with those standing in the open. To confirm convective cooling, we conducted a laboratory study in which radiation, wind speed and ambient temperature were controlled. Finally, we relate our findings to the ecology of this species and in the context of existing knowledge on the convective cooling of some flying insects.

## MATERIALS AND METHODS

### Study area

The research was conducted in the Namib Desert at the Gobabeb Namib Research Institute (23°34′S, 15°03′E) between June 1984 and December 1987. Mean daily air temperature varied across seasons from 18 to 25°C and total daily solar radiation from 14 to 24 MJ m^–2^ (Lancaster et al., 1984; Vogt et al., 2022). Diurnal wind patterns were predominantly calm mornings and cool afternoon sea breezes, except during periodic warm morning ‘berg’ (offshore) winds in winter (Tyson and Seely, 1980).

The activity patterns of free-living beetles were studied in across multiple years at an interdune valley 10 km WNW of Gobabeb. Most of the surface was bare mobile sand, but there were numerous discrete patches of perennial grasses (*Stipagrostis sabulicola* and *Cladoraphis spinosa*) and shrubs (*Trianthema hereroensis* and *Acanthosicyos horridus*) that provided some dappled shade. Detritus patches were concentrated around perennial plants and at the base of dune slipfaces. The valley supported a rich desert fauna with abundant *O. plana*. These beetles, which are usually buried when inactive, were found foraging at detritus patches or searching for mating opportunities. Active beetles were conspicuous as they sprinted between perennial plants or in areas of the dune field devoid of vegetation.

The beetles used in the laboratory studies were collected from the dunes near Gobabeb. In captivity, the beetles were housed on dune sand at air temperatures of 20–25°C. The photoperiod followed that of the Namib environment. A diet of rolled oats, lettuce and apples was provided *ad libitum*. When dead beetles were required for measurements, live specimens were placed into a jar containing cotton soaked in ethyl acetate immediately before experimentation.

### Morphological data

We measured the mass (±0.01 g) of 19 live beetles (9 females, 10 males) using an electronic balance (Mettler AE100, Mettler Instruments, Greifensee, Switzerland). The length and width of the elytra and the dorsoventral depth of the abdomens were measured at the maximum points with dial callipers. The dorsal surface area was measured using a polar planimeter (System Amsler, Zwick GmbH & Co. KG, Ulm, Germany) on 8× magnified photographs of their dorsal surfaces.

### Body temperature measurements

We measured the body temperature of dead and live beetles using fine indwelling thermocouples. A small hole was made with an insect pin (0.45 mm diameter) in the pronotum to the right of the midline, avoiding the dorsal vessel. A copper–constantan (0.08 mm diameter) thermocouple was inserted 3 mm into the thoracic musculature and secured with a drop of cyanoacrylate adhesive. Metabolic heat generated in the prothorax of exercising insects may be transported to, and dissipated from, the abdomen (Heinrich, 1981; Lahondère, 2023). However, the abdomen has a larger surface area subjected to radiation and convection than does the prothorax (Edney, 1971a), and its temperature was significantly higher than the prothoracic temperature in our beetles (paired *t*-tests were significant for every combination of treatment and radiation; Fig. S1A; e.g. at high radiation, standing: *t*=6.375, d.f.=38, *P*<0.001; running: *t*=10.961, d.f.=38, *P*<0.001). Abdominal temperatures, however, were strongly correlated with prothoracic temperatures (*r*=0.976, *t*>11.784, d.f.*=*37, *P*<0.001). So, for live beetles in the field, we used only the probe in the prothoracic musculature as this probe was better secured and less likely to throw running beetles off balance. The fine thermocouple wire was stepped up to a more robust wire (0.4 mm diameter) and connected to a portable digital thermocouple thermometer (Sensortek Inc., Clifton, NJ, USA; calibrated in a vacuum flask against a standard thermometer). Upon completing the measurements, we removed the thermocouple from the beetle and, in the case of live beetles, resealed the pinhole with glue. Neither the implantation nor the removal of the thermocouple had any apparent effect on the live beetles' behaviour, and explanted beetles were found to be active in their habitat on subsequent days.

### Calculations of heat gain

The rate of radiant energy absorbed across the beetle's dorsal surface was estimated from surface area and radiant flux. We assumed that *O. plana* absorbed 94% of the impinging radiation (Henwood, 1975b; McClain et al., 1991; Wang et al., 2021; Willmer and Unwin, 1981) and that other heat balance components (e.g. convection) remained constant. We then calculated the rate at which a beetle's body temperature would rise as a result of exposure to direct downward radiant flux, absorption across the beetle's surface area, its mass and the estimated specific heat capacity of the beetle's tissue:
(1)

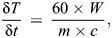
where δ*T*/δ*t* is the rate of body temperature increase (°C min^–1^; where *t* is time), *m* is the average mass (0.77 g for males, 0.91 g for females), *c* is specific heat capacity (3.63 J g^−1^ °C^–1^; Lactin and Johnson, 1998) and *W* is the rate of energy increase (W), which is the product of emissivity (0.94; Wang et al., 2021), surface area (0.000203 m² for males, 0.000193 m² for females) and ambient radiant flux (W m^–2^).

We also estimated the expected rise in body temperature due to metabolic heat production. The oxygen consumption rate in running *O. plana* has been measured for males running at speeds up to 0.2 m s^−1^ (Bartholomew et al., 1985). In walking and running arthropods, the rate of oxygen consumption generally increases linearly with speed (Full et al., 1990; Herreid, 1981). However, anomalously, and importantly, in *O. plana* there was no speed-dependent increase in metabolic heat production at running speeds higher than 0.13 m s^−1^ (Bartholomew et al., 1985), allowing us to extrapolate the rate of oxygen consumption up to 1 m s^−1^. Again, using beetle mass and estimated tissue-specific heat capacity, we calculated the expected rate of rise of body temperature at various running speeds:
(2)

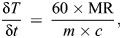
where δ*T*/δ*t*, *m* and *c* are the same as for Eqn 1, and MR is metabolic rate (W):
(3)

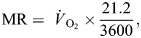
which converts the oxygen consumption rate, *V̇*_O_2__ (ml h^–1^) (Eqn 4), from the oxygen equivalent of carbohydrate combustion of 21.2 J ml^–1^ (Elia and Cummings, 2007) expressed per second:
(4)

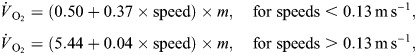
which follows Bartholomew et al. (1985), where *m* is male mass (0.77 g) and ‘speed’ is running speed (m s^–1^ converted from cm s^–1^).

### Activity patterns

Activity patterns of *O. plana* were assessed by walk-through censuses at the field site on three typical winter days (23 April, 7 May and 21 May 1985) and three typical summer days (2, 4 and 5 December 1987). Observers walked two linear transects, aligned north to south, every 15 min over 300 m between 08:45 h and 16:30 h or later in winter, and every 20 min over 400 m between 07:00 h and at least 18:00 h or later in summer. Beetles seen within approximately 12.5 m on either side of the path were counted, so each transect counted the number of beetles in a hectare. The observers were experienced in identifying Namib beetles and could distinguish *O. plana* from other species readily by size, gait, speed and distance travelled, as no other beetle has that unique carriage.

### Measurements of operative temperatures in the field

From a portable meteorological station (CR21 Micrologger, Campbell Scientific Inc., Logan, UT, USA), climatic variables were monitored concurrently with the censuses mentioned above: wind speed, radiation, temperatures of the sand surface and air at 15 mm and 1.5 m above the surface, and black bulb temperature (see Mitchell et al., 2024). A pyranometer (Middleton Instruments, Yarraville, VIC, Australia) was used to measure the ambient downward radiant flux at the field site. We used these microclimatic data to identify an appropriate range of radiant fluxes for calculating radiant heat loads on the beetles and simulating them in the laboratory.

On each day of the walk-through censuses, two dead beetles, one male and one female (*n*=3 pairs; male elytra length 15.5±0.9 mm; female 16.8±0.9 mm) with indwelling prothoracic thermocouples, were placed 15 mm above the sand surface in direct solar radiation, i.e. at the typical body position of running beetles. The temperatures of these beetles were recorded before and after each census. The wind speed at 15 mm above the sand surface was measured using a hot-wire anemometer, recording the mean of six readings over a minute. Temperature excess (*T*_e_) was calculated as the difference between the body temperature of dead beetles and air temperature 15 mm above the sand surface.

### Body temperature of field-active beetles

In 45 field trials conducted in July and November 1984, a live beetle and a recently killed individual of the same sex were held 15 mm above the surface for 3 min to allow body temperature to equilibrate with the ambient temperature. Then, the live individual was allowed to run off while the dead one was left in a standing pose in the sun, 15 mm above the sand surface. At the end of the run, spot measurements of the body temperature of both the runner and the stationary individual were taken. Path distance and running speed were determined with a tape measure and stopwatch.

To measure the body temperature of beetles while active in the field, we threaded a 3 m long thermocouple wire of 0.4 mm diameter through the eyes of a long fishing pole. This wire was stepped down to a 1 m length of 0.08 mm diameter wire inserted into live male beetles (elytra length: 14.6±0.8 mm; *n*=5). An observer held the pole to maintain a slack tether above the beetles, allowing the tethered beetles to move unhindered. The observer followed at a distance to avoid shadowing or influencing their behaviour. Data were collected between 10:00 h and 13:00 h local time, after the beetle temperatures had equilibrated to ambient temperature over a 3 min period. Body temperature was recorded when beetles began to run and 30 s later, and similarly after 30 s of entering shade. Each beetle was tested once only. These measurements were compared with those of recently killed male beetles (elytra length: 15.5±1.0 mm; *n*=5) that were simultaneously placed 15 mm above the sand surface in the sun in a standing pose. From the latter, we could determine how long it took for body temperature to reach threshold temperature when the beetles were not running.

### Heat transfer in the laboratory

**Fig. 3.**
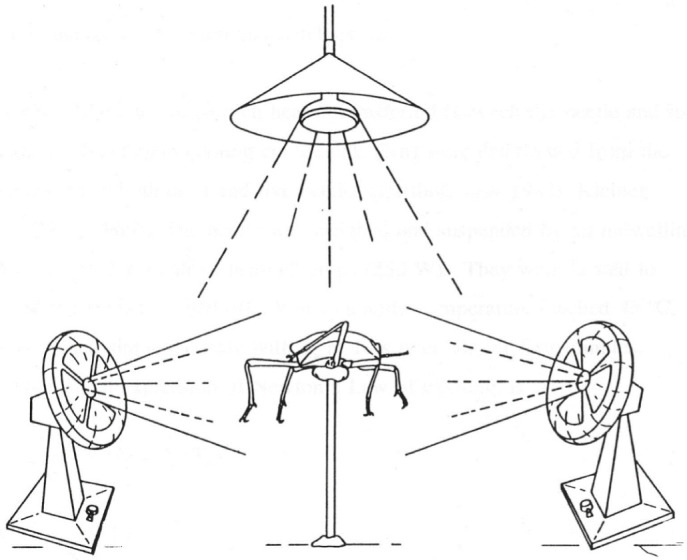
**Solar radiant flux, headwind and ambient wind were simulated in the laboratory.** A beetle (centre) was positioned below a quartz halogen lamp and faced a fan (right) that blew head-on to simulate convection due to running, with another fan (left) blowing side-on to simulate ambient wind.

We tested the effect of forced convection on the temperature of beetles in the laboratory where radiation, wind speed and ambient temperature could be controlled. The body temperature of 18 beetles (6 dead males, 6 live males and 6 dead females) was measured at an air temperature of ∼25°C and that of a further 6 beetles (dead males) at ∼35°C. A capillary glass tube was glued to the ventral surface of the beetle thorax, and the beetle was oriented to face a headwind of 1 m s^−1^ produced by a fan (Fig. 3). This setup simulated the convection produced by a beetle running at its natural speed. A second fan, positioned at right angles to the first, was used to mimic ambient wind speeds of 0 m s^−1^ (calm), ∼0.35 m s^−1^, ∼1.00 m s^−1^ and ∼1.60 m s^−1^. Attaching louvres to the front of each fan ensured a quasi-laminar airflow over the beetle. The orthogonal geometry was chosen to separate maximally the effects of simulated running from those of ambient wind. In the field, beetles did not appear to run at any preferred orientation to the prevailing wind (contrary to Nicolson et al., 1984). Wind speed was measured with a hot-wire anemometer (Alnor Instruments, Shoreview, MN, USA).

A quartz halogen lamp above the beetle provided the radiant heat source. By varying the distance between the lamp and the beetle, three radiant fluxes (∼200, ∼500 and ∼900 W m^–2^), similar to those found at different times during a typical day in the Namib Desert, were generated. The radiant flux was measured with a pyranometer.

A repeated measures design with *n*=6 replicates per group was used to record beetle body temperature at each combination of three radiant fluxes and four transverse wind speeds. At each combination, the equilibrium body temperature of the beetle was determined under conditions of simulated ‘running’ (front fan on, i.e. with head-on airstream) and ‘rest’ (front fan off, i.e. no head-on airstream). Body temperature was considered to have reached equilibrium when the rate of change in body temperature was less than 0.2°C min^−1^. Air temperature measured concurrently with a shaded, white-tipped (Christian and Tracy, 1985) thermocouple (copper–constantan, 0.4 mm diameter) was subtracted from the body temperature to determine the *T*_e_ of the beetle. The difference between *T*_e_ during simulated ‘running’ and that at ‘rest’ was calculated as an index of the cooling induced by simulated running at each combination of radiation and wind speed.

### Data analysis

Data were analysed using Statistica 7.1 (StatSoft) and R statistical software (http://www.R-project.org/). A significance level of 5% was used. Mean values are given ±s.d. Two-sample *t*-tests were used to compare morphometry and *T*_e_ between categories of beetles (male versus female, live versus dead, running versus stationary), while two-way ANOVA were used to test for an interaction effect between radiation level and treatment conditions (simulated running or stationary at different wind speeds). Assumptions of the *t*-tests and ANOVA were checked using boxplots to assess the symmetry of the data in each group (Fig. S1), and Levene's test was used to verify the homogeneity of variance.

## RESULTS

### Morphometrics

There was no significant difference in mean mass or dorsal surface area between males and females, but the mean elytra length, width and dorsoventral depth significantly differed between males and females (Table 1; Table S1). The dorsal surface area was significantly related to beetle mass in males (area=32.39+221.36×mass; *n*=10, *t*=13.278, *P*<0.001) and females (area=68.64+135.65×mass; *n*=9, *t*=5.836, *P*<0.001).

**Table 1.** Morphometrics of live female and male *Onymacris plana* and results of two-sample *t*-tests comparing sexes

	*n*	Mass (g)	Elytra length (mm)	Elytra width (mm)	Dorso-ventral depth (mm)	Dorsal surface area (mm^2^)
Females	9	0.91±0.28	15.7±1.6	13.1±1.6	8.1±0.8	193±40
Males	10	0.77±0.20	14.2±1.2	15.2±2.0	7.1±0.5	203±44
*t*-test		1.2183	2.3764	−2.7139	3.5755	−0.5235
d.f.		17	18	18	18	18
*P*-value		0.2398	0.0288	0.0142	0.0022	0.6070

### Radiant heat gain and metabolic heat production

**Fig. 4.**
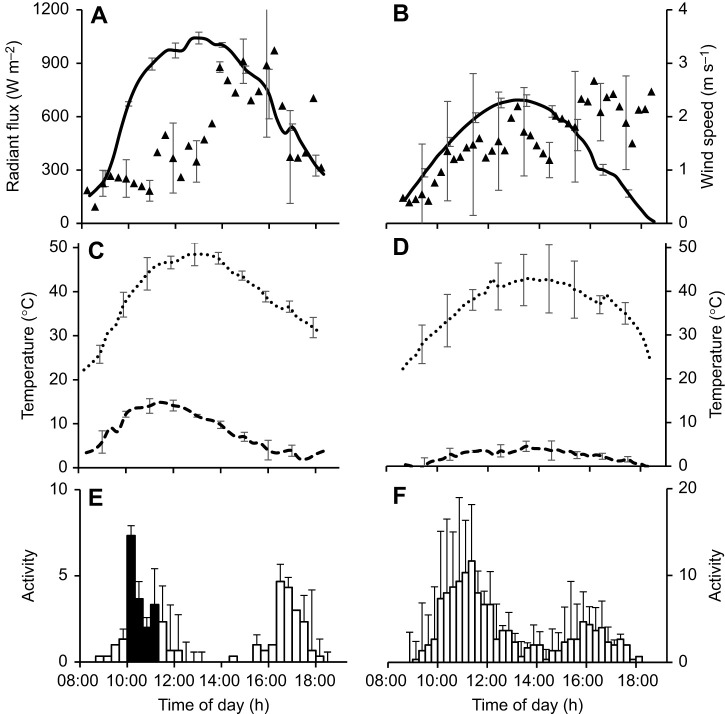
***Onymacris plana* activity and body temperature, radiant flux and wind speed in the field during summer and winter days.** (A,B) Radiant flux (solid curve) and wind speed 15 mm above the sand surface (triangles) on summer days (A; *n*=3) and winter days (B; *n*=3). (C,D) Mean body temperature of dead beetles of both sexes (dotted curve) and temperature excess (*T*_e_) of body temperature above air temperature measured 15 mm above the sand surface (dashed curve) in summer (C) and winter (D). (E,F) Number of beetles recorded being active along dune transects in summer (E) and winter (F). Black bars indicate the 37% of activity during summer conditions of high radiation (>600 W m^–2^) and low wind speed (<1 m s^−1^) when convective cooling by running was effective. Data are means±s.d.

Downward ambient radiant flux peaked at a mean of more than 1000 W m^–2^ on summer days (Fig. 4A) and below 700 W m^–2^ on winter days (Fig. 4B). It exceeded 600 W m^–2^ for over 6 h per day on summer days compared with 3 h per day on winter days.

**Fig. 5.**
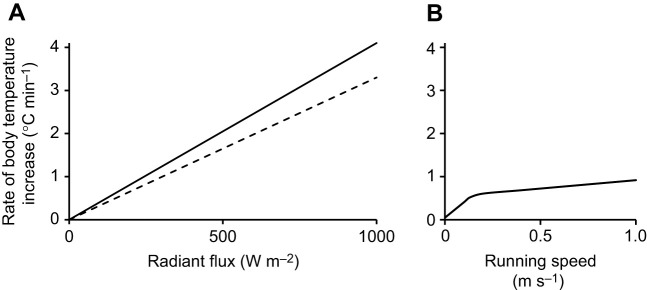
**Predicted rate of increase of body temperature for male and female beetles due to solar radiation and exercise, without body heat dissipation.** (A) Rate of body temperature increase induced in stationary beetles (male: solid line; female: dashed line) by various levels of radiant flux, as calculated from beetle absorption, mass and specific heat capacity, with metabolic rate and other environmental heat transfer assumed constant. (B) Rate of body temperature increase induced by metabolic heat production at different running speeds calculated from oxygen consumption (data of Bartholomew et al., 1985), mass (male) and specific heat capacity, with environmental heat transfer assumed constant.

The rate at which a beetle's body temperature would rise as a result of exposure to radiation was estimated from the flux absorption, beetle mass and estimated tissue-specific heat capacity (Fig. 5A). The predicted rate of rise at peak radiation in summer was 6°C min^−1^ for males and 4°C min^−1^ for females. The expected rate of rise in body temperature due to metabolic heat production at various running speeds was calculated from the rate of oxygen consumption (Fig. 5B). At a running speed of 1 m s^−1^, the expected rate of rise was 1°C min^−1^. So, a beetle running in peak solar radiation would have an expected rate of rise of body temperature of up to 4+1=5°C min^−1^ if it did not dissipate the radiant and metabolic heat. Consequently, beetles starting at a body temperature equal to air temperature, say 25°C, and then running at 1 m s^−1^ in high solar radiation without dissipating radiant and metabolic heat would reach their critical thermal maximum of 50°C in a few minutes.

### Activity patterns and microclimate

The number of *O. plana* active on the surface showed a bimodal pattern on summer days, with the beetles avoiding the most extreme combinations of high solar radiation and low wind speed (Fig. 4; Table S2). Many *O. plana* were active on the surface, though, when radiant flux exceeded 600 W m^–2^ and wind speed simultaneously was below 1 m s^−1^ during their mid-morning activity peak (Fig. 4E). The combined radiant and metabolic heat load on beetles running at 1 m s^−1^ in a radiant flux of 600 W m^–2^ would be expected to cause body temperature to rise at more than 4°C min^−1^ (Fig. 5) if the heat was not dissipated. The beetles tended not to avoid the surface if radiant flux was below 600 W m^–2^, as it was during most of the surface activity on winter days and for much of the afternoon activity period on summer days. In winter, some beetles continued to be active through the hottest times of day, even with radiant flux >600 W m^–2^, a pattern previously recorded for other Namib tenebrionids (Hamilton, 1971; Holm and Edney, 1973; Roberts, 1991; Wharton, 1980).

The *T*_e_ of beetles measured between 08:30 h and 18:30 h in the field differed between stationary dead males and females, with males significantly warmer by 0.4±1.3°C than females in summer (paired *t*-test: *t*=3.0093, d.f.=89, *P*=0.003) and cooler by 0.6±1.3°C in winter (paired *t*-test: *t*=−4.7529, d.f.=109, *P*<0.001). These differences between sexes were overshadowed by the overall diel changes in *T*_e_ by both sexes from 0 to 14.8°C (Fig. 4). *T*_e_ rose quickly in summer, peaking at 14.8°C 2 h before peak radiation and dropping to below 8°C approximately 3 h later (Fig. 4C). This anomaly resulted from the characteristic summer weather pattern (Robinson and Seely, 1980) of cool onshore SW winds picking up around noon (Fig. 4A). As a result of decreased radiation and higher wind speeds during morning hours, *T*_e_ did not exceed 5°C in winter and peaked with radiation (Fig. 4D). As we shall see in the laboratory experiment below, even on summer days, the temperature excess often was in a range such that a headwind of 1 m s^−1^ could eliminate it.

### Body temperature of field-active beetles

**Fig. 6.**
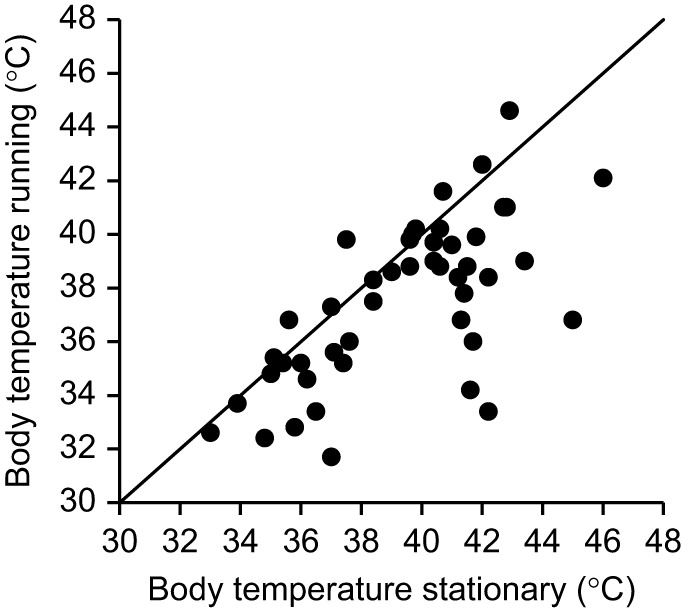
**Thoracic temperature of stationary and running beetles in the sun.** Data are for *n*=46 pairs. The solid line is the line of identity.

Freely active beetles sprinting across dunes (*n*=46) ran at speeds of 0.9±0.2 m s^−1^ (range 0.4–1.3 m s^−1^) for durations of 47±24 s (range 9–131 s), covering path distances of 41±22 m (range 7–109 m). Spot measurements of body temperature at the end of their runs were −1.5±2.5°C (median±s.d.) lower than those of stationary dead beetles with their bodies positioned 15 mm above the sand surface and in the sun (paired *t*-test: *t*=4.9825, d.f.=45, *P*<0.001; Fig. 6; Table S3). Beetles tethered to thermocouples (*n*=5) were −1.5±1.1°C cooler after running for 30 s, which was not different from cooling by standing for 30 s in the shade (−1.4±1.1°C, *t*=−0.040, d.f.=9, *P*=0.9691).

**Fig. 7.**
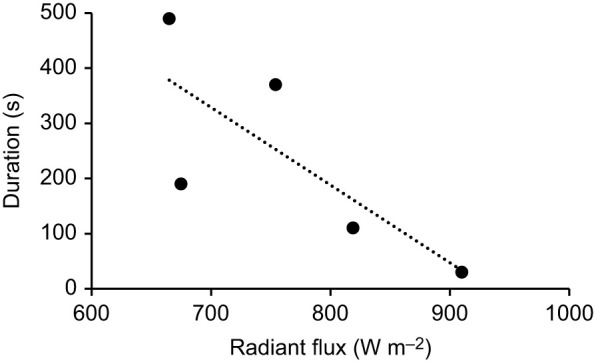
**Duration until the body temperature of five dead beetles placed standing in the sun at high levels of radiant flux reached 46.5°C.** The dotted line shows the regression: duration=1315.301−1.409×radiant flux (*t*=−2.060, *P*=0.1315).

The highest body temperature recorded for a live beetle active in the sun before it sought shade was 46.5°C, well below the species' critical thermal maximum (51°C). When radiation was >600 W m^–2^ in calm conditions in the field, stationary dead beetles reached 46.5°C within 0.5–8 min (Fig. 7).

### Heat transfer in the laboratory

**Fig. 8.**
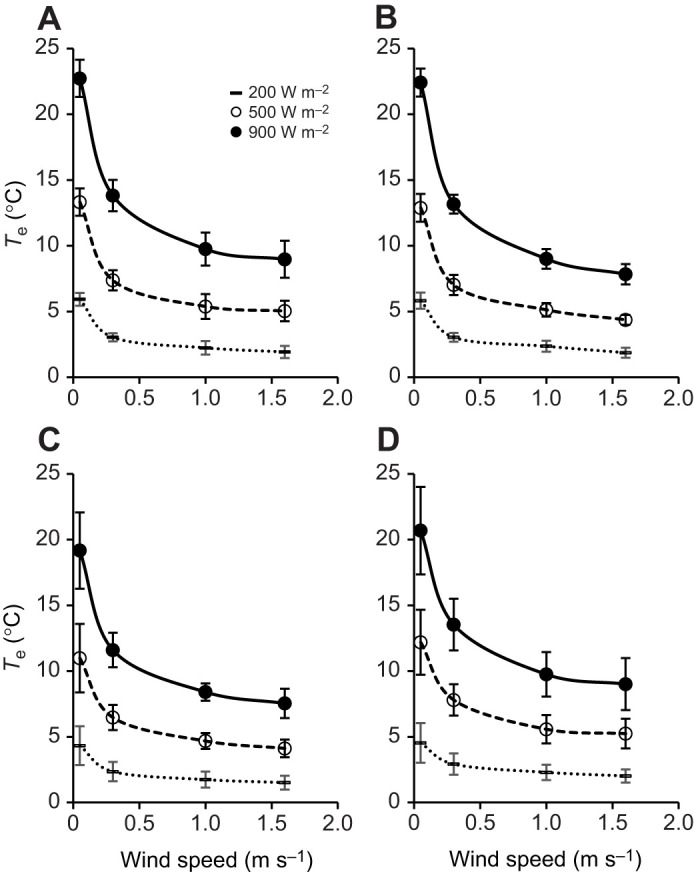
***T*_e_ of *O. plana* in different conditions of radiant flux and wind in the laboratory.** *T*_e_ (mean±s.d.), the difference of body temperature above prevailing air temperature, versus transverse wind speed with a radiant flux of 200, 500 and 900 W m^–2^. The laboratory tests were conducted at an ambient temperature of 25°C with (A) 6 live males, (B) 6 dead males and (C) 6 dead females, and at 35°C with (D) 6 dead males.

A headwind simulating running substantially reduced the excess of body temperature induced by radiation over air temperature (*T*_e_) (Fig. 8). Under controlled laboratory conditions at an ambient temperature of 25°C and radiant flux of 900 W m^–2^, the body temperatures of live beetles exceeded the lethal limit of 51°C without a headwind but only reached a maximum of 39.7°C with a headwind simulating running. Radiant flux, transverse wind speed and simulated running interacted to influence the body temperature of the four groups of beetles (Fig. 8, Table 2). The effect of running was most pronounced when there was no transverse wind and radiant flux was highest. Running had little additional effect on *T*_e_ beyond that induced by transverse winds of ≥1 m s^−1^. A headwind simulating running of live male beetles reduced *T*_e_ by 12.7±1.6°C at high radiant flux and no ambient wind at an air temperature of 25°C (*n*=6; Fig. 8A).

**Table 2.** ANOVA tests of different groups of beetles in laboratory tests with and without a headwind simulating running at different radiation and wind levels

ANOVA	*F*	d.f.	*P*-value	Comparison
2.1*	9.947	2; 30	0.000	Live males (*n*=6) running vs stationary, calm (25°C)
2.2*	0.168	2; 30	0.846	Live males (*n=6*) running vs stationary, wind (25°C)
2.3*	6.969	2; 30	0.003	Live males (*n*=6) stationary, calm vs wind (25°C)
2.4	0.906	2; 426	0.405	Live males (*n*=6) vs dead males (*n*=6) (25°C)
2.5	0.721	2; 462	0.487	Dead males (*n*=6) vs dead females (*n*=6) (25°C)
2.6^‡^	437.5	1; 430	0.000	Dead males 25°C (*n*=6) vs 35°C (*n*=6)
2.7*	7.881	2; 30	0.002	Dead males (*n*=6) running vs stationary, calm (35°C)

*Thoracic temperature was averaged across various wind speeds to avoid pseudoreplication. ^‡^One-way ANOVA comparing thoracic temperature between two ambient temperature groups.

In the two-way ANOVA tests of these laboratory data (Table 2; Fig. S1), there were significant interaction effects between radiant flux and the following variables: running versus stationary in calm conditions by live males at 25°C (ANOVA 2.1) and by dead males at 35°C (ANOVA 2.7), and stationary in wind versus calm by live males at 25°C (ANOVA 2.3). There was also a significant difference in male thorax temperatures between 25°C and 35°C ambient air temperature conditions (ANOVA 2.6) (Table 2).

## DISCUSSION

A diurnally active, apterous, black beetle with a large dorsal body surface area sprinting tens of metres across open terrain in intense sunlight in a hot desert is an anomaly. The radiant heat load on *O. plana*, their morphology and our measurements of radiant heat flux in the desert revealed that they ought not to survive for more than a few minutes before reaching their lethal body temperature of 51°C (Edney, 1971a). They can reduce but not escape (Mitchell et al., 2024) the radiant heat load by seeking shade under the scattered plants in their habitat. However, remaining stationary in a thermal refuge would preclude foraging farther afield, so the beetles do not confine themselves to the shade.

Laboratory studies on the heat transfer of the beetles confirmed the effect of radiation on their body temperature and provided the physical basis of a means by which the beetles could cope with solar radiation. Substrate surface temperature in the Namib Desert frequently exceeds 50°C and reaches 70°C (Curtis, 1985; Holm and Edney, 1973; Lancaster et al., 1984; Lubin and Henschel, 1990; Robinson and Seely, 1980; Viles, 2005), but air temperature 15 mm above the sand surface typically is about 10–15°C cooler than the sand surface temperature during the heat of the day (Marsh, 1985b; Seely et al., 1990). When the beetles run across the dune, their bodies are approximately 15 mm above the surface, and their discoid shape (Fig. 1) ensures that the head, thorax and abdomen are elevated beyond the boundary layer adjacent to the surface. Our estimates of the effects of radiation on the body temperature of the beetles would have been underestimates, because we calculated downward radiation only, and there would have been some longwave radiation from the hot sand surface to the undersurface of the beetles (Mitchell et al., 2024).

In the laboratory, we simulated both the convective cooling of running and the convective cooling of ambient wind. The simulations revealed that the body temperature rise induced by simulated solar radiation could be counteracted by subjecting beetles to a headwind equal to their running speed. A headwind of 1 m s^−1^, not statistically different to the remarkably constant running speed of 0.9±0.2 m s^−1^ that we measured in 46 beetles, reduced the body temperature of beetles subject to radiant flux up to 900 W m^–2^ much closer to air temperature. Transverse ambient wind also significantly reduced body temperature increase due to the radiant heat load, such that the cooling of simulated running provided no additional benefit at transverse wind speeds of 1 m s^−1^ and higher. That the body temperature, measured by Nicolson et al. (1984), of beetles that had just completed a sprint across the dune base was not different to that of resting beetles was to be expected; the ambient wind speed during their study was 5–7 m s^−1^.

Our laboratory measurements provided a physical explanation for our observations of body temperature in the field. When beetles leave the shade to cover territory exposed to solar radiation, one would expect their body temperature to increase, especially when ambient wind speed is low. One would expect their body temperature to increase further if they ran faster, as a result of metabolic heat production (Heinrich, 1981), even though *O. plana* has a lower metabolic rate during exercise than expected, allometrically, for an arthropod of its mass (Fleming and Bateman, 2007). We calculated that the rate of body temperature rise of beetles running in the sun could be up to 7°C min^−1^ (Fig. 5). However when we measured the body temperature of 46 beetles at the end of sprints in the sun, their temperatures were 1.5°C lower than those of dead beetles in standing postures in the sun. Because the effect of air movement on convective and evaporative cooling is identical (Mitchell et al., 2018), in theory, the beetles could have cooled evaporatively while running. However, the low rates of water loss of tenebrionid beetles, even for desert insects (Zachariassen et al., 1987), diminish any role of evaporation in the cutaneous cooling induced by running. Evaporative water loss through respiration would have been increased when metabolic rate increased during running but is a minor contributor to heat balance in *O. plana* (Duncan, 2021; Edney, 1971b). We therefore conclude that convection is the primary method of cooling that is enhanced by running. Notably, most beetles were active during periods of lower solar load and higher wind speeds (Fig. 4), even when they could have been stationary in shade.

Female *O. plana* beetles had a lower ratio of surface area to mass than did males, partly due to their rounder shape (Henwood, 1975a). Consequently, the rate of radiant heat gain was lower for female beetles, and forced convection had a lower cooling effect (Henwood, 1975a). Therefore, the male beetles heated up and cooled down more quickly than did the females. They also had a greater temperature excess above air temperature in calm, high radiation conditions in summer, but this excess was lower in winter, perhaps because their smaller dorsoventral depth reduced their exposure to the lateral rays from the lower winter sun. We cannot predict whether female beetles can dissipate enough heat by running to lower their body temperature, as no data on their metabolic rate are available. If the aerodynamic form of male beetles is responsible for the low cost of locomotion, as Bartholomew et al. (1985) suggested, female beetles may have a higher energy expenditure during running than do males.

Although, as far as we know, no one previously has reported exercise-induced cooling in terrestrial locomotion, body temperature did not rise in the large dung beetle, *Heliocopris dilloni*, while walking in still air in the laboratory without radiation (Bartholomew and Heinrich, 1978). It is possible that some fast-running ants, which are active during hot times, also cool convectively. In hot desert conditions, with surface temperatures exceeding 50°C, Marsh (1985a) recorded *Ocymyrmex barbiger* running at speeds up to 0.4 m s^−1^, on a par with *Cataglyphis bicolor* in similar conditions (Tross et al., 2021; Wehner, 1994). Like *O. plana*, *Ocymyrmex* and *Cataglyphis* have long legs facilitating sprinting and stilting (Sommer and Wehner, 2012), and high critical thermal maxima, up to 55°C in *C. bicolor*, among the highest of all terrestrial invertebrates (Gehring and Wehner, 1995; Marsh, 1985a,b; Roberts et al., 1991).

Exercise-induced cooling has been recorded in insects flying at high speeds. Indeed, the body temperatures of flying insects tend to depend more on their capacity to cool during flights than on the rate of metabolic heat production induced by exercise (Heinrich, 1981). The tiger beetle *Cicindela tranquebarica* flies when temperatures on the ground become too high; its low wing load and low wingbeat frequency generate little metabolic heat, and the increased convective cooling during flight causes a decrease in body temperature (Morgan, 1985). When flying in conditions of high solar radiation, the desert bee *Centris pallida* cools by doubling its convection and transferring heat from the thorax to the abdomen (Johnson et al., 2025).

Male blue-black grassquits, *Volatinia jacarina*, of Central America can engage in an energetic aerial courtship display even at hot times of the day because the display dissipates as much heat convectively as it produces metabolically (Weathers, 1986). The wingbeats of male hoverflies, *Syrphus* sp., may generate sufficient convective cooling to compensate for metabolic heating (Gilbert, 1984). Brazilian free-tailed bats, *Tadarida brasiliensis*, had lower rectal temperatures at the end of flights than at the beginning, losing heat by radiation to the night sky faster than they generated it metabolically (Reichard et al., 2010). Famously, the comic-book flying elephant Dumbo was allegedly able to dissipate all his metabolic heat through his ears while flying (Phillips and Heath, 2001).

### Conclusions

Running speed and body morphometrics enable *O. plana* to be active on the sand surface during thermally stressful periods. Being able to move about in adverse conditions is advantageous as the wind-blown detritus, their primary food source, occurs in small isolated patches on dunes. Mobile beetles can forage before the shifting sands cover the food source. They can be active when their predators and other animals sharing the same habitat and food source cannot be active. Also, males can prolong their search for mates reproducing aseasonally. We predict that beetles will eventually slow down and reduce convective cooling with ageing because abrasion of the tarsal tips results in shorter legs and less traction (Enders and Schüle, 1993).

Although one might suppose that thermophiles would be at an advantage with global warming, a meta-analysis by Weaving et al. (2022) found that the adaptation of critical thermal maxima is not keeping pace with rising ambient temperatures. As a result, the thermal windows that allow surface activity between dawn and dusk will eventually shrink for thermophiles. In the Namib Desert, this curtailment of thermal windows will be exacerbated by the predicted increase in aridity due to climate change (Engelbrecht et al., 2024), which will reduce the shade-providing vegetation and food availability required to fuel exercise.

## Supplementary Material

10.1242/jexbio.250379_sup1 Supplementary information

Table S1. Onymacris plana body measurements and radiant heat exchange (see Results: Morphometrics)

Table S2. Onymacris plana activity census, prothoracic temperature and microclimate (see Results: Activity patterns and microclimate)

Table S3. Spot measurements of prothoracic temperatures of O.plana after running compared to stationary (dead) beetles (see Results: Body temperature of fieldactive beetles)

Table S4. Laboratory simulations of running or standing of O.plana under different conditions of radiation and wind (see Results: Heat transfer in the laboratory)
