# Supplementary material for: Beetling the heat – the diurnal Namib Desert beetle Onymacris plana cools by running
Source: J Exp Biol. 2025 Aug 14;228(16):jeb250379. doi: 10.1242/jeb.250379 (PMC12401538; doi:10.1242/jeb.250379)
Supplement: Supplementary information [file jexbio-228-250379-s1.pdf]

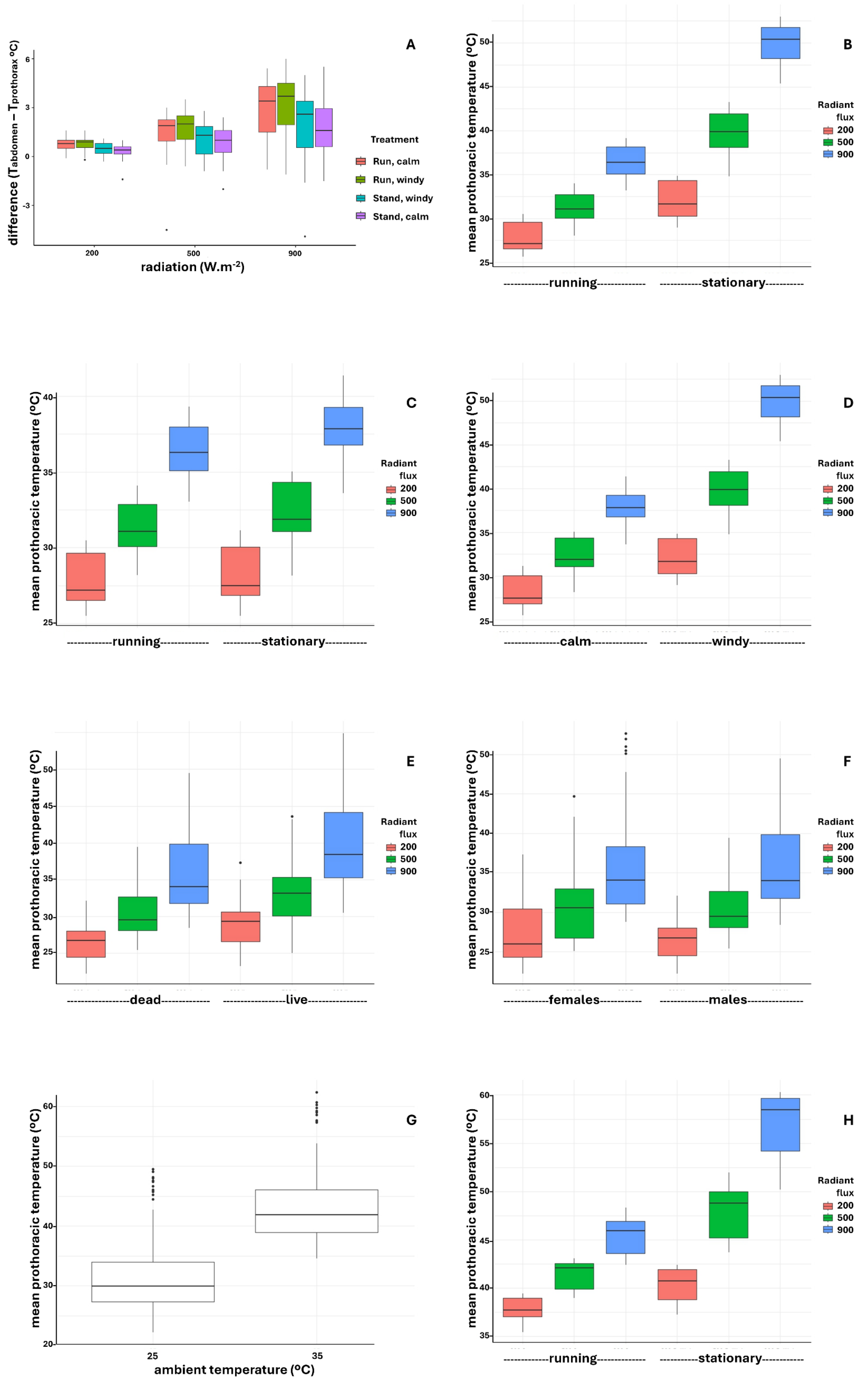

Journal of Experimental Biology • Supplementary information

Fig. S1. Box plots of prothoracic temperatures of Onymacris plana in laboratory tests in different radiant fluxes. (A) Difference between prothoracic and abdominal temperatures, (B) live males running vs stationary without wind at an ambient temperature of 25ºC, (C) live males running vs stationary with wind at 25ºC, (D) stationary live males without vs with wind at 25ºC, (E) live males vs dead males at 25ºC, (F) dead males vs dead females at 25ºC, (G) dead males at ambient temperature of 25ºC vs 35ºC, (H) dead males simulated running vs stationary without wind at 35ºC.

Table S1. Onymacris plana body measurements and radiant heat exchange (see Results: Morphometrics)

Available for download at
https://journals.biologists.com/jeb/article-lookup/doi/10.1242/jeb.250379#supplementary-data

Journal of Experimental Biology • Supplementary information

Table S2. Onymacris plana activity census, prothoracic temperature and microclimate (see Results: Activity patterns and microclimate)

Available for download at
https://journals.biologists.com/jeb/article-lookup/doi/10.1242/jeb.250379#supplementary-data

Table S3. Spot measurements of prothoracic temperatures of O.plana after running compared to stationary (dead) beetles (see Results: Body temperature of field-active beetles)

Available for download at
https://journals.biologists.com/jeb/article-lookup/doi/10.1242/jeb.250379#supplementary-data

Table S4. Laboratory simulations of running or standing of O.plana under different conditions of radiation and wind (see Results: Heat transfer in the laboratory)

Available for download at
https://journals.biologists.com/jeb/article-lookup/doi/10.1242/jeb.250379#supplementary-data

Journal of Experimental Biology • Supplementary information
